# Medical Transactions, Published by the College of Physicians in London. Vol. V

**Published:** 1816-10

**Authors:** 


*Medical Transactions, published by the College of Physicians in London. Vol. V.*

( Continued from page 137. )

The first Part of the Analysis of this Volume was terminated by our plucking a few feathers from the wing of Mr. Millington. [See page 137.] It is somewhat unfortunate that this gentleman did not recollect the fable of the poor bird doomed to expiate a fit of presumption by the loss of its plumes. Thus might he have been spared the torture of a feathering; and ourselves the pain, little less severe, of inflicting it. But to speak seriously, we think the honour and dignity of the College of Physicians compromised by the admission of a paper which the Editors of at least two of the three Medical Journals published in London, would, we are convinced, certainly have rejected.

*XVI. Some Cases illustrative of the Pathology of the Brain.* By Richard Powell, M. D. &c.—Notwithstanding the labour and attention with which it has been long cultivated, the pathology of the encephalon is, at the present hour, less perfectly understood than that of any other important organ of the animal economy. The intricacy of its structure, and the deep obscurity in which its functions are involved, tend powerfully to aggravate the difficulties of the research. Upon this subject, the present labours of Dr. Powell are laudably directed. His paper contains thirteen interesting cases; in eleven of which, the external morbid phænomena were clearly elucidated by dissection. One of the most instructive we shall select for quotation. The general outline of the others we can only allow ourselves to trace with the utmost brevity.

A Peruvian by birth, and soldier by profession, who had suffered much anxiety, was attacked, two years before death, by severe pains in the head. They gradually subsided, leaving a constant sense of tightness across the forehead, and of " want of room within the skull." Eight months afterwards, the vision of the right eye was suddenly diminished, and at length completely lost. The left shared the same fate. In this state of blindness, the effect of emetics was very remarkable.

" In the act of vomiting, the power of vision was suddenly restored to the right eye, with a sensation as if a flash of lightening had taken place, but it remained only for the short space of an hour, the clear vision gradually subsiding to a glimmering of light, and at last becoming extinguished."

The pupils were fully dilated, the retinæ quite insensible. After the lapse of a year, he applied for relief of gastric derangement, signalized by uneasiness of the stomach, anorexia, and disposition to vomit. The bowels were inactive; urine copious; pulse natural; tongue pale-coloured. The tightness of the forehead continued. The body was sluggish; the intellect clear and vigorous. Medicine did not relieve the nausea: weakness and constipation ensued. Emetics were again tried. After the second, the iris regained, and preserved during life, its mobility, but vision was not restored. Impending apoplexy was averted by local abstraction of blood, and blistering. The patient became sleepy; his articulation indistinct. The mental faculties remained clear, the sense of hearing remarkably acute, to the last. *Dissection.*—The arachnoid membrane on the upper part of the brain was considerably thickened. The ventricles contained an unusual quantity of water with a little " matter floating in it." The pituitary gland, enlarged to five times its natural size, was changed into a pulpy structure; the sella turcica proportionately enlarged. To the upper part of the gland was connected an oval tumour, large as a hen's egg, which occupied the site of the infundibulum, separated the optic nerves, extended into the third ventricle, and contained a thick purulent fluid. The fibres of the optic nerve were expanded, and almost destroyed, towards the tumour. The vessels of the brain were unusually turgid. The plexus choroides was thickened, and contained some small earthy concretions.

The results of the other eleven fatal cases will be most clearly and concisely exhibited by the following table. The history which we have cited, forms the eighth in Dr. Powell's series.

### Cases.

| *Antecedent morbid Phænomena.* | *Morbid Appearances on Dissection.* |
|---|---|
| 1. Stupor, insensibility, general convulsions; pupils dilated. Eruption on the hands recently repelled by topical applications. Subject a female; age 17. | None discovered in the brain or abdomen. |
| 2. Instantaneous death preceded by slight head-ache. | Effusion of blood into the ventricles of the brain. |
| 3. Head-ache, convulsions, insensibility, in a boy, aged 8. | Meningeal vessels of the brain loaded: two ounces of bloody water effused into the lateral ventricles. |
| 4. Severe diarrhœa, mania, fatuity; urine and stools passed involuntarily; convulsions affecting the left side of the body more than the right, and causing death. An adult male. | A firm, thick, vascular membrane, beneath the dura mater, and enveloping the right hemisphere of the brain. The arachnoid beneath, thickened, and inclosing a gelatinous fluid. Two ounces of water in the ventricles. |
| 5. Deafness after scarlatina, suppuration in the right ear, following measles; violent pain: little constitutional derangement: drowsiness, rapid exhaustion, death. A boy, aged 16 | Coagulable lymph and pus covering the right hemisphere of the brain, and collected between the posterior lobe and tentorium. Petrous portion of the temporal bone carious: dura mater above it detached and sloughy. |
| 6. Convulsive affection of the left side; right side weak; health little disturbed. Sudden hemiplegia of the left side, with aphonia and stertor; right arm convulsed. Coma, insensibility, death. A male, aged 70. | Arachnoid and pia mater opaque; eight ounces of aqueous fluid effused between the membranes, and into the ventricles of the brain. Ulceration in the fore-part of the anterior lobe of each hemisphere. |
| 7. Hysteria, head-ache, imperfect vision, pupils dilated: convulsion, transient aphonia; utter loss of sight.—Intellect clear; abdominal functions deranged; insensibility. A female, aged 23. | Cerebral membranes loaded: Four tubercles imbedded in the right hemisphere, which was unnaturally large. One tubercle in the left hemisphere, less developed. Aqueous fluid accumulated in the ventricles. |
| 9. Head-ache, stupor; intellect disturbed; pupils dilated; articulation indistinct. Hiccough, right eye utterly insensible. Inconsciousness; paralysis of the right side. A male, aged 20. | Four ounces of aqueous fluid in the ventricles; effusion of coagulable lymph on the base of the brain. Small white tubercles originating from nearly the whole of the inner surface of the pia mater. |

### Cases.

| *Antecedent morbid Phænomena.* | *Morbid Appearances on Dissection.* |
|---|---|
| 10. Violent periodic head-ache; vision double during a severe paroxysm; twitching and numbness of the left side of the body; vision permanently double; convulsions of the right side. An adult male. | A tumour anteriorly in the right hemisphere of the brain, very firm towards the centre, and compressing the right ventricle. Half an ounce of water in the left ventricle. |
| 11. Violent head-ache, at first periodic, afterwards constant; vision weakened, particularly that of the left eye; left side of the face rather drawn; restlsesness, delirium; vision gradually lost. Fatal apoplectic seizure. A male, aged 30. | A tumour on the base of the anterior lobe of the left hemisphere of the brain; the medullary substance of the hemisphere reduced to a pulpy state; the cortical sound. Some fluid contained in the ventricles. |
| 12. Hæmoptysis; restlessness, mental disturbance more violent during night; coma by day. No pain of the head. A male subject, aged 48. | Right sac of the pleura containing much pus: Liver enlarged and morbid. A large cyst, inclosing pus and blood, beneath the anterior part of the corpus callosum, and projecting into the right ventricle. |

Dr. Powell terminates his "gloomy catalogue" by the narration of a case in which abstraction of blood from the temporal artery, during a dreadful paroxysm, was productive of signal benefit. His recommendation of arteriotomy in affections of the head, requiring depletion, we can, from experience, most cordially second; and frequently have we regretted to see a less effectual operation preferred to it, from some vague and groundless apprehension of its attendant difficulties. That man, call himself what he will, is no surgeon, who would hesitate or blunder in the incision of the temporal artery.

The value of this communication is, in our view, greatly enhanced by considering the rank and situation of its writer. Fellows of the College, too commonly, like the fastidious *Fellowes of the army*, cherish a contempt for the drudgery of the scalpel, sublime and dignified in their own conception, as disgraceful, mean, and despicable in ours. Highly then are we gratified to see such zeal for the cultivation of morbid anatomy evinced by a man of Dr. Powell's character and talents. May the influence of his example among the medical students of St. Bartholomew's, be deep and extensive, as his exertions and their object are honourable! May they be taught to consider clinical and anatomical observation as the two eyes of physic, each reciprocally aiding—each imperfect without, the other; and a blind physician, like a profligate priest, as one of the deepest curses that society can foster within its bosom.

*XVII. Remarks on Palpitations, and on Epilepsy.* By THOMAS YOUNG, M. D. F. R. S. &c.—From the well-known effect of a watery fluid in transmitting pulsatory motions, Dr. Young attempts to explain the anomalous appearances sometimes accompanying palpitations of the heart; and which, improperly viewed, give rise to mistaken notions on the physiology and pathology of the sanguiferous system. We shall select, in illustration of his doctrine, the first of two cases which he records. A middle-aged woman, after repeated attacks of acute rheumatism, was seized with palpitation of the heart, several times recurring. Violent pain about the navel succeeded. Respiration became laborious. Dropsical effusion into the abdomen and cellular membrane repeatedly yielded to diuretics, and the pain was much relieved; but always during the presence of it and the abdominal swelling, the complexion was yellow, the lips and nails purple. Palpitation was observed in the heart, in the *right* hypochondriac region, and *right* side of the neck. The pulsation of the latter was unconnected with the stroke of the carotid artery, and not confined, Dr. Yonng *asserts,* to the jugular vein. The patient could not recline on the left side. She was once more greatly relieved by medicine; but the dropsical symptoms again shewed themselves. The palpitations of the heart, neck, and abdomen, were much diminished: the motion of the former organ, however, was fluttering; the pulse intermitting and irregular. Weakness and diarrhœa succeeded. At length, the dropsy nearly subsided, and the palpitation was, in great measure, confined to the heart. The general symptoms were again somewhat relieved by recurrence of the rheumatic affection of the joints; and the woman soon after died. On *dissection,* the heart was found enlarged; *the valves of the pulmonary artery so much ossified as to impede the free passage of the blood;* an accumulation of fluid in the pericardium, in the right cavity of the thorax (probably about *twelve ounces),* and in the ventricles of the brain: "little or none" in the left thoracic cavity; the liver dark-coloured. Now Dr. Young, resting upon these facts, attempts to prove that the pulsatory motion, displayed on the right side of the neck and in the right hypochondrium, arose from the action of the heart, propagated by the effused fluid of the corresponding thoracic cavity to those regions. The obvious difficulty, resulting from the small quantity of that fluid, evidently inadequate to such effect, he surmounts by the *supposition,* that as the general dropsical symptoms had subsided, a great portion of the effused fluid might have been removed from the thoracic cavity. This argument derives, in his opinion, additional force, from the decrease of pulsation of the neck, noted on the decline of the dropsical swellings; and the absence of pulsation on the left side is explained by the immunity of the left thoracic cavity from the effusion. The inference attempted to be established is, that pulsation in the neck and hypochondrium may be regarded as "an additional nosological test applicable to the discrimination of dropsy of the chest."

But to this hypothesis of Dr. Young, many and formidable objections present themselves. In the first place, the accuracy of the Doctor's premises is exceedingly suspicious: for, granting that the diminution of fluid in the right cavity of the chest were absolute fact, instead of mere conjecture as it now is, its admission must open the door to another difficulty. Twelve ounces of fluid were poured out into the right cavity of the chest, and a "little" into the left.* Now, if we allow that a considerable portion of such fluid had been taken up from the former, is it not possible, nay probable, that the latter also might have been relieved in like manner? unless Dr. Young can ingeniously contrive to exclude it from the rights and privileges of the absorbent process. And be it remembered, that, even when the heart is *not* "enlarged," the left pleura is always less capacious than the right. Again, whenever any fixed obstacle exists to the passage of the blood through the pulmonary cavities of the heart, pulsation of the *right* cervical and hepatic regions is frequently, and without any complication of hydro-thorax, observed. This phænomenon we have been accustomed to refer to the consequent reflux of blood into the superior vena cava and

* "Little or none" is a vague mode of expression, highly improper in scientific description, and unworthy of Dr. Young. Either there was fluid, or there was not. But the very expression indicates its existence. Why then was not the precise quantity correctly ascertained and specified? Does not Dr. Young consider percussion of the chest as a "nosological test"—a mean of discrimination, applicable to cases of hydrothorax? If so, why did he not employ it, and state the results?

right jugular,* into the inferior cava and hepatic veins, on each contraction of the cavity situated anteriorly to the obstructed orifice, in the route of circulation. In a case of cardiac lesion, so precisely resembling that which forms the subject of our present criticism as to require no description, we ourselves, some time since, observed a similar phænomenon. The sonorous condition of the thorax, at first, bespoke its cavities free from effusion. Universal dropsy supervened. The sound of the chest became gradually obscure. Every other sign of hydrothorax was distinctly marked. The pulsations of the neck and hypochondrium *declined as the accumulation rose.* This we attributed to the oppressed state of the heart, and its diminished powers of re-action, consequent either on the load of thoracic fluid, or on the exhaustion of the vital energies, perhaps both. No medicine would excite to activity the absorbent system. The unfortunate woman perished from suffocation. After all, it cannot be denied, that there is much ingenuity, and perhaps some truth, in the explanation of Dr. Young. All we contend for is, that anomalous pulsations of the neck and abdomen may exist, as in the case at which we have just glanced, independently of hydrothorax; and, as in the example cited by himself, admit of explication unconnected with the phænomena of hydrostatics. Hence, in the present state of our knowledge concerning them, these pulsations cannot be legitimately regarded as a pathognomonic sign of dropsy of the chest.—The second case is rather inimical, than otherwise, to Dr. Young's hypothesis. The cervical pulsation existed on both sides. An accumulation of serum was found in the pericardium, but *not in the sacs of the pleura.* The heart was considerably enlarged, "particularly the right auricle:" but whether it was thickening of the parietes, or dilatation, of the cavity, we are not informed. That, on accurate examination, it would have been found to be the latter, complicated with obstruction in the orifice of the right ventricle or of the pulmonary artery, we will venture to affirm. The dissection was performed by a hospital *pupil.*—The paper is concluded by a recital of two *cases of epilepsy*, apparently unconnected with worms; which both yielded to *oleum terebinthinæ* once largely ad-

* Why the *right* jugular vein will, in these cases, exhibit the phænomenon of pulsation more frequently or more decidedly than the *left*, they, who understand the disposition of the system of vessels of the superior cava, will not require to be informed. Rev.

ministered. Thus employed, we think that it probably operates on the same principle as terror, intoxication, or other moral or external agents, which, by exciting a sudden and violent commotion in the system, have been known permanently to remove the epileptic paroxysm. Dr. Young has derived no benefit from the exhibition of small doses of turpentine in epilepsy. When the disease originates from organic lesion within the cranium, this, like all other stimulant remedies, is evidently contra-indicated.

*XVIII. Extracts from a Paper on Phthisis, by the late* M. ORBAN, Surgeon in the French Navy. Translated from the French by the REGISTRAR.—New and successful remedies for pulmonary consumption may delight the visionary, or impose upon the credulous and inexperienced. Far different are the feelings with which we contemplate them. They pass athwart our sober vision, like the meteor rushing to extinction; frail and perishable as it; and excite only a painful recollection of the many fair delusions which the passing brightness has heretofore tempted us to indulge, and of the ravages which this "Dæmon of the West" has committed among the friends and associates of our early years. An insulated ulcer of the lungs, whether arising from inflammation of the bronchial membrane, rupture of a blood-vessel, or deep-seated suppuration, may, and does sometimes, even under circumstances apparently hopeless, admit of cure; and all the fortunately terminating cases of *advanced* phthisis will, we are convinced, if their history is correctly traced, be found to belong to one of these three species: but that recovery can be permanently established, when the substance of the lungs is studded by tubercles in a state of suppuration, it would require more confidence in the powers of nature or of art, than we profess, to credit. We have heard, indeed, of such things as the cure of confirmed tubercular phthisis by this man's plaster, or that man's pill; but, amid the crowds of victims annually hurrying to the grave under our own observation, we have not yet seen one solitary instance of it. The number, the unkindly nature and secretion of the ulcers, their inaccessibility to any direct application, the impossibility of excluding from them the atmospheric air, or obviating its noxious influence, and lastly, of preserving the morbid lungs in a state of quietude, constitute, unfortunately, a chain of circumstances through which the arm of science, however ably directed, will never break; and whereby the more common and destructive species of phthisis is placed, like gastric scirrhus and thoracic aneurism, far beyond the reach of all its boasted resources.—But it is time to say something of M. Orban's plan of treatment. This consists in administering one pill, with four ounces of an acidulous drink, nine times a day.* He first derived it from the practice of a Moorish physician, of Tunis, upon one of whose patients, far gone in *catarrhal* phthisis, he witnessed its successful operation. The Moor restricted the invalid to two meals a day: they consisted of boiled millet or vermicelli, without liquid. Constipation of the bowels he considered as a most auspicious symptom, which he never attempted to obviate. Nine cases of phthisis are recorded by the French surgeon, in which this plan, with slight modifications, was instituted. Six of these are *said* to have been permanently cured by it.

*XIX. A Case of Elephantiasis.* By EDWARD ROBERTS, M. D. &c.—The subject of this horrible affection was a boy, aged fourteen, by birth an American. It first attacked the head and face after exposure to rain during three successive nights, upon his voyage to England. Some slight indisposition previously suffered, the head, eye-lids, and lobes of the ears, began to enlarge, and tubercles appeared on the face. Breath offensive; urine thick; genitals shrunk; hair fallen from the pubis and eye-brows; sexual desire extinct. The tubercles, after landing, extended to the hands and feet. The countenance was much disfigured; lips swollen; alæ nasi enlarged, flattened, scabrous; the disease at length extending to the throat; the gums tumid and vascular; voice weak and hoarse. The tuberculated parts differed little in colour from the rest of the skin, and were insensible. There was a troublesome itching, with issue of a sanious fluid from some parts, succeeded by a scab: on some, superficial ulceration took place; from others, a "furfuraceous scaling of the cuticle." With the exception of head-ache and total absence of perspiration, the lad's health was little disturbed. After the unavailing

* The pills are thus formed: Take of benzoic acid half a scruple; alum, sulphate of iron, gum arabic, each scrup. j; soot, a handful. Levigate them with water on a marble, and form them into seventy pills. The liquid consists of rain water, oz. xlij; white wine vinegar, oz. vj; refined sugar, oz. ij. The twelve ounces of this mixture, not consumed with the pills, were administered warm during the night. M. Orban, in some cases, substituted citric for acetic acid; and always excluded the *iron* from the pills.

trial of various remedies, he got completely well under the employment of generous diet, cinchona-decoction, and small doses of submuriate of quicksilver and opium internally; and of vapours of hot water to the face, and warm fomentations to the extremities. The cinchona was prescribed for a herpetic affection of the abdomen, complicated with fever. Diarrhœa and cough came on during recovery. The lad, after a residence of eight months, was discharged from St. Bartholomew's, with an "entire new countenance."

*XX. Case of Puerperal Fever, in which the Uterus and Spleen were principally affected.* By HUGH LEE, M. D. &c.—A woman, aged 28, in whom a severe attack of puerperal fever, occurring on the fourth day from delivery, had been subdued by venæsection, blistering, and purging, was found, after a week of apparent convalescence, during which, however, obstinate constipation had prevailed, in a very alarming state. There was much constitutional irritation, without pain or other symptom of local affection. Abdomen painless on pressure; skin hot, but clammy; pulse rapid, small, compressible; respiration frequent, anxious, laborious; countenance jaundiced, and cheeks exhibiting the circumscribed redness of hectic; tongue foul, little thirst, urine tinged with bile, and bowels obstinately costive. Strong cathartics, and subsequently cordials, were administered in vain. The woman sunk rapidly, and died in a few hours.

On *dissection,* were found slight turgescence of the peritoneal vessels; sigmoid flexure of the colon inflamed, and adhering to the left altered Fallopian tube; uterus slightly inflamed externally; its vessels enlarged and turgid, internal surface exhibiting a gangrenous, irregular appearance, dark, livid, or greenish, with adherent portions of coagulated blood resembling polypi; left ovary much diseased, vagina of a livid colour; spleen adhering to the adjacent peritonæum; its internal structure soft, spongy, and filled by an intimate mixture of pus and grumous blood. The two kidnies, united at their inferior extremities, "formed one continuous body of a semi-lunar form." In structure, they were healthy. The unnatural formation of these organs is illustrated by a coarse and spiritless engraving. The observations of Dr. Lee possess little interest. He thinks that the phænomena, observed on the internal surface of the uterus, did not arise from gangrene, but from decomposition of the effused blood and lymph. He farther speculates on the connexion of inflamed spleen with puerperal fever, and quotes the writings of the French Gastellier, in proof of its frequency.

XXI. *Observations respecting the Safety and Efficacy of the internal Use of Superacetate of Lead in Pulmonary Consumption.* By JOHN LATHAM, M.D. &c.—The learned writer of this paper finds a difficulty in persuading himself that lead is "necessarily detrimental to the animal body." Indeed, he considers it destitute of the "deleterious qualities almost universally assigned to it." At least, whatever destructive properties lead itself may possess, "nothing pernicious can be charged upon it in the superacetated form;" and it is "agreeable to other analogies" to "*suspect*" *that the lead*, "*although in itself actually poisonous, becomes innocuous by the addition of acetous (acetic) acid.*" These are the words of Dr. Latham; and nothing can exceed the astonishment with which we read them. Is it possible, that the President of a British College of Physicians can be ignorant of facts with which every student of medicine must be familiar? *Lead, in its metallic state, is perfectly inert; and only acquires deleterious properties in its various combinations with oxygen or its compounds.* However this be, Dr. Latham recommends the superacetate to be given in "very large quantities, not only in hæmorrhages, but in colliquative diarrhœas and hectic perspirations, and more especially in that semi-purulent expectoration which too often terminates in pulmonary ulceration and consumption." He has even seen it "effect a cure," where there has been a "very copious expectoration of actual pus." He commonly prescribes it in combination with opium, and has never known mischief result from its employment. But we would warn the young practitioner not to confide implicitly in such sweeping and unqualified statements. The acetate of lead is indeed a valuable medicine, and may sometimes be largely administered, not only with impunity, but with excellent effect: yet, we have occasionally witnessed violent symptoms produced by the ingestion of comparatively slight doses. It is curious, that every paper contributed by Dr. Latham to this volume, has, for its main object, an eulogium on some preparation of *lead*. These saturnine propensities in the head of the College are surely somewhat alarming. We hope that Saturn is not its presiding deity!

XXII. *A Case of Fever, attended with inordinate Appetite.* By R. P. SATTERLEY, M. D. &c.—Anorexia is almost an invariable concomitant of fever. But, in this case, the patient, a boy of sixteen, became, on the fifth day of an attack of severe typhus, ravenously hungry; would consume at a meal, without being satiated, "a pound and a half of beef steaks, a large fowl, or a couple of rabbits;" and, moreover, eat many pounds of dry bread, biscuits, or fruits, during the day. The voracious appetite always began with the febrile paroxysm, and declined with it. During the remission, he slept soundly. Restriction with respect to food produced great distress, and aggravated the febrile symptoms. Substances, the most hard and incongruous, were greedily swallowed; and when every thing else failed, he would attack the bed-clothes, and even bite his own fingers till they bled. Digestion was unimpaired. By the assistance of strong purgatives, an enormous quantity of solid natural fæces was evacuated; but no watery stools. After thirty days, the fever declined, and the patient gradually recovered.

XXIII. *Three Cases of convulsive Affection.* By RICHARD POWELL, M. D. &c.—"My object in the relation of these cases has been chiefly to check by them, the account I formerly gave of the effects of the nitrate of silver." Every one will agree with Dr. Powell, that it is "as useful to record instances of failure in the effect of remedies as of their success." The cases, described, are all those of chorea, although probably differing in source, and modified in character, by the influence of age, sex, and other circumstances of the patients.

*Case* 1. A tailor, aged 37, suffered five years ago, a singular convulsive affection, and is said to have lost his senses during twelve days. Nitrate of silver, *in doses of fourteen grains every four hours,* was given for a month, and subsequently opium, aconite, arsenic, zinc, and purgatives, without effect. Camphor removed the disease. In the second attack, occurring in 1815, oil of turpentine, exhibited to the amount of one ounce, induced purging and faintness, and was succeeded by "uniform and constant trembling rather than occasional decided convulsions." Belladonna, cinchona, nitrate of silver, opium, assafœtida injections, and the shower-bath, were tried in vain. The affection again yielded to camphor, administered in doses of from ten to fifteen grains every four hours.

*Case* 2. A boy, aged 6, suffering a second attack; the convulsions general, but stronger on the right side than on the left; abdomen large and painful; fæces offensive. The disease was not mitigated by five grains of nitrate of silver, given every four hours; but soon yielded to repeated doses of calomel and scammony, which unloaded the bowels and restored their secretions to a healthy state. This, Dr. Powell observes, is not the only instance in which the application of the precepts of Dr. Hamilton "has led to the cure of the disease, after the methods I am more in the habit of previously employing have failed."

*Case* 3. A girl, aged 17. The origin of the convulsions attributed to terror: bowels rather slow; fæces dark. After the operation of a mercurial purgative, one ounce of oil of turpentine was given: its effects violent. Increased convulsion, delirium, vomiting, and purging, were the prelude to a calm. The menstrual flux now first shewed itself; and the convulsions gradually subsided. Nitrate of silver and the cold bath were used to expedite convalescence. Dr. Powell suggests the expediency of trying oil of turpentine in *hydrophobia.* This paper stands honourably distinguished by the spirit of candour in which it is written.

*XXIV. Facts exemplifying the Efficacy of the Cow-pox, in preventing and mitigating the Malignity of the natural Small-pox.* By George Sandeman, M. D. &c. The title of this paper sufficiently illustrates its import and value. We pass on to a more fruitful and interesting subject.

*XXV. Some Observations concerning the Fever which prevailed at Cambridge during the Spring of* 1815. By T. Haviland, M. A. Professor of Anatomy in the University of Cambridge.

*XXVI. A Statement of Two Cases of Fever which occurred at Cambridge.* By R. Harrison, M. D. &c.— Every medical practitioner of this island has, doubtless, heard of the Cambridge fever, and formed his own opinions as to its source, character, and treatment. On these subjects, wide difference of sentiment unfortunately existed among the gentlemen, within whose sphere of observation the malady broke out; and remedies equally dissimilar, in many cases, distinguished their practice. Far removed from the scene of pestilence and dispute; utterly unknown to, and unconnected with, any person whose opinions may become the subject of criticism; unbiassed, consequently, by interest or prejudice, we shall minutely and dispassionately analyse the two interesting papers before us, and attempt to draw from the data afforded by them, inferences which may elucidate the origin and nature of the malady; and tend to establish, on the sure basis of induction, a plan of treatment, as decisive as it is clearly indicated.

For the sake of perspicuity, we shall arrange our description under the three distinct heads of *History of the Disease, Pathology,* and *Treatment.* We shall conclude with such observations as a review of the subject may suggest, and such inferences as the facts, which it exhibits, may legitimately sanction.

*History.* The fever first shewed itself in Cambridge, about the commencement of February, 1815. Its attacks, with few exceptions, were confined to persons below the middle age. The children of the poor were particularly obnoxious to it. No doubt of its infectious nature can exist: for Professor Haviland states, that a servant, who went home ill from Cambridge, communicated it to "nearly all" her family: it proved fatal to her father. Other instances of a similar nature have been reported both to the Professor and ourselves. It seems to have possessed a local origin; for some time elapsed ere it invaded, and then only in a partial manner, the surrounding villages. Professor Haviland is "disposed to attribute it," and we conceive with perfect accuracy, "to the state of the drains and ditches which have been much neglected of late, whilst the population has greatly increased."— As a standard of description of its general characters and course, we shall select the first case detailed by the Professor. A female servant (age not mentioned) on the *fifth* day of an attack, which had not till then rendered her incapable of work, exhibited the following symptoms: great debility, languor, head-ache, anorexia, white tongue, frequent pulse, full and costive bowels.—*Sixth day.* Pyrexia and head-ache aggravated; delirium impending. —*Seventh.* Active and violent delirium, with propensity to suicide; little variation for some days.—*Tenth.* Delirium continued; pulse 120; skin hot and parched; great thirst. On the preceding night, she had escaped into the street, and rolled in the kennel, in order, as she said, "to cool herself." — Morning of the *twelfth.* Extremities cold; pulse fluttering; great exhaustion. The delirium aggravated by employment of a little wine. The morbid heat of the surface never returned. From this time till the *seventeenth* day, when death took place, progressive aggravation of the symptoms; pulse varying from 120 to 130; *tongue dry and brown;* delirium unabated; bowels irregular, with disposition to costiveness; tremor and subsultus, without, however, any great actual debility of the muscular system, for the woman could stand, unsupported, almost to the last. It could not be ascertained that she had been exposed to the agency of infection. Professor Haviland farther observes, that the onset of the disease was, in general, slow and insidious; that the bowels were, in most cases, deranged, commonly costive; their discharges dark, unhealthy, and offensive; and that increased heat of the surface was not an invariable attendant, a sensation of cold, perceptible to others, being sometimes complained of by the patient. In the two cases recorded by Dr. Harrison, the head was at first affected with dizziness; the appetite continued all along unimpaired, and convulsions came on. The vision of the elder brother, who recovered, was considerably disordered, and repeated bleeding exerted no evident influence on his "banging pulse."

*Pathology.* On inspection of the body of a gentleman, aged 19, who fell a martyr to the disease, Dr. Harrison states that the following morbid appearances were discovered: vessels of the dura mater distended with blood; other cerebral vessels unnaturally full; a large quantity of fluid effused between the dura and pia mater; many "blotches of blood" contained in the medullary substance of the brain; lateral ventricles very much distended with fluid. The gall-bladder empty; the intestinal canal much distended with flatus. Thorax not examined.* What sound and obvious lessons does this interesting dissection inculcate!

*Treatment.* In the first and only fatal case detailed by Professor Haviland, the remedies were a purgative at first, repeated blisters, antimonials, cold applications to the head, cold affusion towards the close, wine and light cordials. In a second case, aperients, blister to the neck, and *leeches to the temples,* followed by the most signal relief and speedy recovery.—Third case. An emetic, which operated on the bowels, and *leeches to the temples.* The

---

* The same dissection, at least we judge it to be so, both from the correspondence of the initials and other circumstances, is detailed somewhat differently by Professor Haviland. We have thought proper to avail ourselves of Dr. Harrison's description. The difference is not considerable.

head-ache and tendency to delirium were thereby prevented or removed, and the fever continued its progress, unaccompanied by these formidable symptoms, and consequently, in great measure, divested of its dangers. The treatment, afterwards generally adopted by the Professor, was clearing the stomach and bowels by purgatives and antimonials; counteracting the determination to the head by leeches and sometimes blisters; exhibiting saline medicines and mineral acids, with which were combined, "as soon as the symptoms would admit," the lighter preparations of cinchona. Wine was sometimes useful, but could only be borne in small quantities. The cold affusion did no good in the case first related; and in another, wherein it was accidentally used, a relapse was the consequence of its employment: but in the former, it was *first* had recourse to on the *tenth* day; in the latter, during a state of perspiration! Large doses of purgatives were rather prejudicial than otherwise, by inducing intestinal torpor and flatulence. We question, however, whether, *on the first onset of the malady*, they were administered in a sufficiently vigorous form and dose. Cinchona, if prescribed too early, induced considerable derangement of the bowels. The decoction and tincture were found adequate to "complete the cure." So much for the observation and evidence of Professor Haviland. A detail of the *early* treatment of his first and fatal case is very properly suppressed by Dr. Harrison from feelings of delicacy to the relatives of the deceased. All we know is, that blisters had been applied about the ninth day of the disease, just previously to Dr. Harrison's *first* visit; but *no abstraction of blood, local or general, had been then practised.* Dr. Briggs, who, on the *fifteenth*, took charge of the case, directed the application of *leeches to the temples*, and blisters to the legs and head; and the patient, so far from having his debility increased, *was considerably relieved by the evacuation.* Cordials were afterwards administered. Death took place on the seventeenth day. The results of the dissection we have already stated.

The subject of the other case, a robust young man, aged twenty-four, was first attacked about the close of March. April 2d. He took a calomel purgative, and lost *sixteen ounces of blood* from the arm. By leeches, lancet, and cupping, *sixty more ounces were abstracted at five different times, from the 10th to the 26th of April, without affecting the pulse*, or inducing great muscular debility. The other remedies employed were purgatives and diaphoretics, with

*low diet:* afterwards, *submuriate of quicksilver, in repeated doses,* opium, cinchona, blisters behind the ears, occasional purgatives, wine. About the middle of June, after a severe struggle, convalescence advanced rapidly. The blood drawn on the 18th was "*very buffy.*" Local bleeding was productive of greater relief than venæsection.

Such are the facts which we glean from a calm and dispassionate review of the statements of Dr. Harrison and Professor Haviland relative to the Cambridge fever, and they derive ample confirmation, direct or indirect, from all that we have, elsewhere, heard or read respecting it. The inferences, which may be rigorously deduced from them, are next to be examined. These, if we mistake not, are the following:

That the fever, which broke out at Cambridge, in the commencement of 1815, was inflammation of the brain, complicated with fever, either essentially typhoid, or at least, extremely prone to degenerate into typhus;

That it was, at first, of local origin, though afterwards, in many instances, propagated by infection; and that it probably may be referred to the noxious influence of the effluvia of animal and vegetable substances in a state of decomposition;*

That its fatal issue was, for the most part, determined by the unsubdued inflammation of the cerebral organ, and consequent effusion between the membranes, or into the ventricles; but that, when this inflammation was in itself mild, or checked, or arrested by proper evacuations, the disease then commonly ran on and degenerated into absolute typhus;†

That the remedies, correctly indicated in the first stage of the disease, were such as would speedily and effectually relieve the increased action and turgescence of the vessels of the brain, *particularly local and general abstraction of blood;* but that when, from the neglect of these remedies, or some peculiarity of the fever itself, such increased action had been suffered to exhaust itself, or induced a dangerous state of debility, and the characteristic

---

* Some years since, we saw, in a medical student of St. Bartholomew's, a disease precisely similar, both in its external phænomena, and in the appearances on dissection, to the first case recorded by Dr. Harrison. It was evidently induced by exposure, for several hours, *to the emanations of a putrid body in the dissecting room.*

† The third case, which we have noted as occurring in the Practice of Professor Haviland, will constitute a clear illustration of this fact.

signs of pure typhus were developed, a plan of treatment, calculated to support the declining energies of the system, could alone be had recourse to with prospect of success.

These views will, we conceive, reconcile much of the discrepancy of opinion, much of the contradiction in practice, unfortunately exhibited by the gentlemen on whom the treatment of the Cambridge fever devolved. Drs. Harrison and Briggs, in denying, on the one hand, the existence of all complication of typhus; and Mr. Okes, in characterizing it, on the other, as essentially and primarily a fever of debility, appear, we think, to have erred in the opposite extremes. The views of Professor Haviland, as to the *nature* of the malady, are certainly by far the most correct. We wish that, in the *treatment,* he had evinced somewhat more of the energy and decision which signalized the practice of Dr. Harrison. Yet, had the latter gentleman pushed the calomel more freely in the *earlier* stages of his second case, recovery, which seems, in a great measure, attributable to the operation of that remedy, would probably have been less wavering and protracted. In any case where effusion had decidedly taken place, mercury must evidently take the lead of the few remedies which would then constitute the physician's " forlorn hope."

Lastly, we may be allowed to observe, in confirmation of the opinions just delivered on the nature of the Cambridge fever, that typhus is invariably accompanied by signs of increased action in one or other of the more important organs* of the animal œconomy, most frequently of the brain. Sometimes, however, this local affection is so slightly marked as to elude the eye of the hasty and superficial observer. But is the typhus of this country ever known to run its course, unattended by some degree of head-ach, vertigo, confusion, or stupor, more or less strongly marked? Does the brain of persons perishing from typhus, ever fail to exhibit traces of increased vascularity and increased action? If the correctness of our clinical and anatomical observation may be relied on, certainly not! Struck by the close coincidence of these

* What are pneumonia typhodes, typhus icterodes, and puerperal fever, but inflammation of the lungs, of the hepatic system, and of the uterus and its investing peritoneum, complicated with typhus? and are not these diseases, for the most part, dreadfully infectious?

phænomena, some able pathologists have boldly asserted that typhus fever is essentially dependent on chronic inflammation of the brain or its membranes. We wish not to enter into controversies, for the decision of which a sufficient number of facts has not yet been accumulated. All we contend for is, that few cases of typhus, in the worst and severest forms under which it, at present, visits this island, occur; wherein low diet, brisk intestinal evacuants, and *abstraction of blood by leeches from the temple,* may not, *on the first onset,* be prescribed with decided benefit. Where the patient is robust, and the characters of the malady are such as to admit of it, we prefer incision of the *external jugular vein:* for thus the advantages of local and general bleeding are combined in one operation.

*XXVII. The History of a Case of Purpura Hæmorrhagica.* By G. D. Yeats, M. D. &c.—The symptoms of this disease, occurring in a young woman of eighteen, were oozing of florid blood from the mouth and nose, without previous indisposition; gradual and general eruption of spots on the skin, not elevated, and greatly varying in size; loss of appetite; aching and stiffness of the calves of the legs; pain at the stomach; skin cool; pulse 80, firm and full; no thirst; countenance pale; bowels torpid. Sickness and vomiting afterwards came on, succeeded by alarming debility. The blood continued to flow, but less frequently; and the spots, from which it had issued, became enlarged and dark coloured. On the *seventh* day, menstruation took place, and the blood formed a *coagulum.* This Dr. Yeats supposes to arise from deficiency of secreting action in the uterine vessels, whence the blood passed through them, unchanged; and he considers the observation valuable, as illustrating by analogy, that inaction of all the extreme vessels which characterizes this disease, and may "readily be conceived," to be the "immediate cause of the petechiæ and hæmorrhage." The remedies prescribed, were saline aperients, mineral acids, cinchona, wine, ablutions with warm vinegar, the effervescing saline draught with opium. As the internal administration of cinchona disordered the stomach, two ounces of it, finely powdered, were quilted in a calico jacket and applied round the waist. Dr. Yeats had twice before tried this mode of exhibiting cinchona with success. His patient, in this case, recovered gradually after the eighth day. An ingenious attempt is made to explain the "extreme quickness (rapidity?) and smallnes of the pulse,"

the "fainting and prostration of strength," which, on this occasion, manifested themselves. They appear, says the Doctor, "to have arisen from the quantity of blood which was thrown out of the circulation by effusion under the skin, producing petechiæ and vibices." And he farther adds: "As soon, however, as the extreme arteries began to recover their healthy action, and to transmit to the corresponding veins, instead of letting it escape into the cellular membrane, the blood they received, the pulse became fuller and slower, and gradually returned to its healthy state."

*XXVIII. History of a Case of Somnambulism.* By the SAME.—This affection, like all others of a similar nature, was evidently connected with deranged function of the chylo-poietic viscera. After obstinate resistance, it yielded to remedies, the object of which was, to improve the state of the digestive organs, regulate the action of the bowels, and tranquillize the system. Sufficient attention is commonly not paid to diet in these cases. Dr. Yeats very judiciously remarks, that, in all continued derangements of the digestive process, the efforts of the physician should not be exclusively directed to the stomach. The conditions of the liver and duodenum ought also to be vigilantly regarded. Many bilious affections, and supposed liver complaints, have, we verily believe, their real seat in the important duodenal portion of the intestinal tube.

*XXIX. Farther Observations on the Treatment of Pulmonary Consumption.* By E. ROBERTS, M. D. &c.—Recommendation of the Moorish plan of treatment in phthisis is the object of Dr. Roberts. Three cases are brought forward, in which the acetic acid was administered with great and lasting benefit: but the symptoms are so imperfectly detailed, that we can hardly ascertain whether the disease were in reality pulmonic phthisis, or what the precise species to which each case belonged. In several instances, the acetic acid is acknowledged to have failed. It was generally prescribed by Dr. Roberts in half ounce doses, combined with infusion of cascarilla and a little syrup. In a Postscript, referring to his paper on the employment of nitrate of silver in painters' colic, the Doctor recommends, that this active mineral should be directed in *solution* only, as he has twice seen intestinal hæmorrhage result from its exhibition in a *solid* form.

*XXX. A Case of Tetanus.* By WALTER VAUGHAN, M. D. &c.—A healthy boy, aged 9, had the first joint of his little finger shockingly crushed. On the sixth day from the accident amputation was performed. Tetanus declared itself on the tenth. He was first seen by Dr. Vaughan on the fifteenth. He was much better on the *following* day, and speedily recovered. The compound powder of ipecacuanha was given in *small* repeated doses; submuriate of quicksilver with scammony, *once.* The stump was fomented with warm milk and water, and afterwards poulticed. Dame Nature, we imagine, did more than the Doctor in this case; or verily she must have been in one of her most desperately idle fits!

*XXXI. Signum mortis pathognomonicum in homine repentinâ morte extincto, innocuâ infallibilique operatione confirmatum, haud ita pridem inventum a* FRANCISCO ROMERO, M. D. Gotholaunico, &c. &c.—Although man and death have been so long and familiarly acquainted, and much of the attention of physiologists has been directed to the curious and interesting phænomena which signalize the ravages of the destroyer; commencement of animal decomposition is, perhaps, the only positive criterion by which extinction of the vital principle can, at present, be ascertained. Hence many a poor wretch has been consigned, by ignorant, thoughtless, or interested attendants, to the horrors of premature interment. To discover a test whereby cessation of life may be *immediately* recognized; to ascertain the precise point at which the empire of light terminates, and that of darkness begins, becomes then an object of as deep importance to the sciences of medicine and legislation as to humanity itself. Such a test, as simple and easy of application as it is "infallible," the learned foreigner asserts that he has long known and practised; and the communication of it to the College forms the purport of his paper.

"If, in a few moments from the introduction of a small feather, moistened with concentrated ammonia, into the nostrils of a person who has made an expiration unusually deep, a light froth appears, it is a pathognomonic sign of death."

The cause of this phænomenon is unknown. The author farther recommends the application of *moxa* immediately under the head of each fibula, where the external cutaneous nerve passes down superficially. Should no trace of vitality be exhibited on this operation, the subject of it may be considered as certainly dead. Dr. R.

has, sixty times, repeated this experiment within eighteen years; and three persons, in whom the pathognomonic sign did not appear, were recovered by the application of proper means. In all the others, it was invariably seen. We are admonished to exercise great vigilance and attention while making the experiment: "for the phænomenon is transient, and vanishes in a moment." This paper is written in the Latin language: its style is somewhat too courtly and florid for a scientific production.

Here our task terminates.* May we be allowed to express a hope, humble as sincere, that the term of intellectual gestation of the learned body, whose labours we have just been scrutinizing, may be reduced from twelve years to as many months; and that the next brat, equal in size to its brethren, may have a little more flesh upon its bones: in other words, that the Royal College of Physicians in London may publish, *annually*, a volume quite as *large* as their fifth, and *much more closely printed!*

* By a mistake either of the Reviewer or Printer, the number of papers was stated, in the former part of our analysis, to be *thirty-five* instead of *thirty-one*.

